# Surgical outcomes of major vascular resection for retroperitoneal liposarcoma from a high‑volume sarcoma center: a propensity score matching analysis

**DOI:** 10.1007/s00432-024-05871-7

**Published:** 2024-07-09

**Authors:** Guoqiang Xue, Xiaopeng Wang, Bonan Liu, Chengpeng Li, Ang lv, Xiuyun Tian, Jianhui Wu, Hui Qiu, Chunyi Hao

**Affiliations:** Key Laboratory of Carcinogenesis and Translational Research (Ministry of Education/Beijing), Sarcoma Center, Peking University Cancer Hospital & Institute, Beijing, 100142 People’s Republic of China

**Keywords:** Retroperitoneal liposarcoma, Vascular resection, Major complication, Mortality, Survival

## Abstract

**Purpose:**

Radical resection of retroperitoneal liposarcoma (RLPS) may necessitate vascular resection and reconstruction. The study was conducted to assess surgical outcomes of surgery for RLPS with major vascular involvement.

**Methods:**

Patients with RLPS who underwent surgical resection at the Sarcoma Center of Peking University Cancer Hospital between April 2011 and December 2022 were identified from a prospectively maintained database. Patients were classified into two groups: vascular resection and non-vascular resection groups. A propensity score matching analysis was performed to eliminate baseline differences between the groups. Surgical details and postoperative outcomes were analyzed. Furthermore, prognostic factors for local recurrence-free survival (LRFS) and overall survival (OS) were assessed.

**Results:**

Overall, 199 patients were identified and the median follow-up period was 48 (interquartile range [IQR] 45–69) months. Vascular resection was performed in 42 (21%) patients, 25 of whom had vascular infiltration. A total of 39 patients had vascular replacement and 3 patients underwent partial resection (side-wall resection). Vascular resection was burdened by higher rates of major morbidity (38% vs. 14%, *p* < 0.001) and 30-day mortality (7.1% vs. 1.3%, *p* = 0.005). After propensity-matched analysis, patients who underwent vascular resection had 5-year LRFS and OS rates comparable to those without vascular involvement. Major vascular resection was not an independent risk factor for LRFS or OS.

**Conclusions:**

Although accompanied by increased risks of major morbidity and mortality, the major vascular resection enabled radical resection in patients with advanced RLPS, affording comparable 5-year LRFS and OS rates compared to those who did not.

## Introduction

Retroperitoneal sarcomas (RPS) are heterogeneous entities of retroperitoneal mesenchymal origin, accounting for approximately 15-20% of soft tissue sarcomas (Schmitz and Nessim 2022). The main histological type of RPS is retroperitoneal liposarcoma (RLPS) (Danieli et al. 2023). Although the role of neoadjuvant and adjuvant therapy in RPS has not been validated definitively, radiotherapy and chemotherapy can be considered for highly selected cases as indicated on the NCCN guideline. (de Bree et al. 2023; von Mehren et al. 2022). Radical excision is the cornerstone of therapy for improving survival among patients with RLPS (Improta et al. 2023).

Considering the wide potential space of the retroperitoneum, RLPS usually presents as a large mass and is prone to encase or abut vital viscera and vessels (Danieli et al. 2023). However, radical resection with the involved major vessels is often challenging (Tzanis et al. 2018). While tumor resection with the involved vessels increases the risk of postoperative complications, previous analyses have shown it can improve local control (Schwarzbach et al. 2006). Given the advancement in operative techniques and perioperative management, major vascular involvement does not represent a contraindication for the curative resection of RLPS (Devaud et al. 2023). Comparable survival rates have been reported between patients who underwent vascular resection and those who did not (Blair et al. 2018). However, most reports of major vascular resection for RPS are based on small series or heterogeneous tumors with various subtypes (Devaud et al. 2023).

Due to the rarity of RLPS, histology-tailored outcomes of surgery with major vascular involvement have yet to be well established. This study focused on patients with RLPS and aimed to investigate surgical outcomes of major vascular resection by using a propensity score matching analysis.

## Materials and methods

### Patient population

All consecutive patients who underwent curative-intent resection for RLPS between April 2011 and December 2022 at the Peking University Cancer Hospital Sarcoma Center were retrospectively reviewed. None of the patients received neoadjuvant and adjuvant chemotherapy or radiation in this study. The inclusion criteria were as follows: (1) complete clinical and follow-up information, (2) histologically confirmed liposarcoma, (3) located in the retroperitoneum, (4) underwent macroscopically complete (R0/R1). Patients who lost to follow-up (*n* = 3) and received macroscopically incomplete (R2) (*n* = 25) were excluded. Finally, 199 consecutive patients were included in the study.

The study was approved by the ethics committee of the Peking University Cancer Hospital. Patients provided written informed consent for data collection.

### Surgical management

Diagnosis and treatment for patients with RLPS were planned at weekly multi-disciplinary treatment (MDT) meetings including oncological surgeons, medical oncologists, radiologists, pathologists, radiation oncologists, and nuclear medicine specialists. Based on the preoperative imaging, extensive multi-visceral resection of the tumor and adjacent organs was rational for tumors with potential resectability even if not overtly infiltrated, as recommended in the consensus manuscripts by the Transatlantic Australasian Retroperitoneal Sarcoma Working Group (TARPSWG) (Lv et al. 2022). At our institution, all operations were performed by the same surgical team without vascular surgeon collaboration. As one of the largest specialized referral sarcoma centers in China, the team has considerable expertise in surgical oncology and is capable of performing major abdominal operations, including hepatectomy with inferior vena cava resection and reconstruction, pancreaticoduodenectomy with superior mesentericoportal venous resection, total pelvic exenteration, and major vessel resection and reconstruction. Vascular resection was performed as follows: encasement, involvement or vascular occlusion (Spolverato et al. 2021). Major vascular resection can be partial or complete. When a primary anastomosis was impossible, we favored the use of polytetrafluoroethylene (PTFE) prosthesis to restore continuity.

### Diagnosis and definition

RLPS was diagnosed by two specialized sarcoma pathologists. The following characteristics of RLPS patients were collected from a prospectively maintained database: age, gender, Body Mass Index (BMI), presentation status (primary or recurrence), histological subtypes, size, French Federation of Cancer Centers Sarcoma Group (FNCLCC) grade, multifocality, the number of organs resected, pancreatic resection, nephrectomy, and major vascular resection.

Patients were prospectively followed every 3–4 months for the first 2 years, every 6 months for 3 years, and yearly thereafter (Baia et al. 2023). Clinical examinations, and abdominopelvic CT/magnetic resonance imaging (MRI) were performed during follow-up. Clavien-Dindo (CD) grade and postoperative mortality within 30 days were collected. The CD grade III or greater was considered “major” (Dindo et al. 2004). Local recurrence-free survival (LRFS) was defined as the time from surgical resection to the onset of recurrence or the last follow-up. Overall survival (OS) was defined as the time from surgical resection to the last follow-up or death.

Pathological subtypes were classified based on the 2020 World Health Organization criteria (Kallen and Hornick 2021). The 3-tiered grading system of FNCLCC was applied to sign tumor grade (Neuville et al. 2014). The multifocality was defined as the presence of two or more noncontiguous neoplasms. Surgical resection was classified as macroscopically incomplete (R2) or complete (R0/R1) (Gronchi et al. 2012). Major vascular resection was defined as the resection the following vessels: the celiac axis, aorta, portal vein, superior mesenteric vein, inferior vena cava, or external/common iliac vein/artery (Tzanis et al. 2018). BMI was calculated as weight (kg)/height (m)^2^. The number of organs resected, operative time and estimated blood loss were determined based on the operative report documented by the surgeon. The graft patency was evaluated on the contrast-enhanced computed tomography (CT) scan imaging after surgery.

### Statistical analysis

Results were expressed as medians with interquartile ranges (IQR) for continuous variables and numbers with percentages for categorical data. Chi-squared test and Mann–Whitney U test were performed to compare categorical and continuous variables, respectively. Patients were classified into 2 groups: those who underwent major vascular resection and those who did not. A propensity score analysis was performed to overcome biases between the two groups. Propensity scores were estimated using a 1:2 “nearest neighbor” matching method with a caliper width of 0.05. After matching, univariable and multivariate Cox proportional hazards analysis was used to explore the effect of various parameters on survival, which were reported as Hazard Ratio (HR) and 95% confidence intervals (95%CIs). Survival curves were estimated using the Kaplan–Meier method and compared using the log-rank test. A two-sided P value of less than 0.05 was considered statistically significant. Statistical analyses were performed using SPSS version 26.0 (Chicago, IL, USA) and R software (version 4.3.1; http://www.r-project.org/).

## Results

### The baseline characteristics

Of the identified 199 patients, 42 patients underwent major vascular resection (Table 1). For patients who underwent vascular resection, the median age was 54 years and 45.2% were female. The predominant histological subtype was dedifferentiated (66.7%), followed by well-differentiated (21.4%), pleomorphic (7.1%), and myxoid/round cell liposarcoma (4.8%). 20 patients (47.6%) presented with recurrent disease and multifocality was found in 33.3% of the patients. The tumor location was identified as follows: abdominal (*n* = 22, 52.4%), abdominopelvic (*n* = 17, 40.5%), and pelvic disease (*n* = 3, 7.1%). A high FNCLCC grade (G2 or G3) were found in the majority of the tumors (83.3%). The median size of the sarcomas was 23 (IQR 13–30) cm and the median number of organs resected was 6 (IQR 4–8). Patients who underwent vascular resection were more likely to experience increased number of organs resected (*p* = 0.032), and have abdominopelvic tumors (*p* = 0.043). After matching, all baseline characteristics between the two groups were comparable (*P* > 0.05 for all).

**Table 1:** The baseline characteristics before and after propensity score matching

Variables	Vascular resection (*n* = 42)	Before matching		After matching	
		Non-vascular resection (*n* = 157)	*P* value	Non-vascular resection (*n* = 84)	*P* value
Gender			0.881^a^		1.000^a^
Male	23 (54.8)	88 (56.1)		46 (54.8)	
Female	19 (45.2)	69 (43.9)		38 (45.2)	
Age (yr) [ median (IQR)]	54 (47–60)	58 (49–64)	0.090^b^	57 (49–62)	0.221^b^
BMI	23.7 (21.1–25.8)	23.6 (21.5–25.8)	0.886^b^	23.8 (21.9–26.5)	0.611^b^
Presentation status			0.342^a^		0.704^a^
Primary	22 (52.4)	95 (60.5)		47 (56)	
Recurrence	20 (47.6)	62 (39.5)		37 (44)	
Size(cm) [ median (IQR)]	23 (13–30)	20 (14–28)	0.332^b^	21 (14.6–28)	0.395^b^
Location			0.043^a^		1.000^a^
Abdominal	22 (52.4)	113 (72)		44 (52.4)	
Abdominopelvic	17 (40.5)	37 (23.6)		34 (40.5)	
Pelvic	3 (7.1)	7 (4.4)		6 (7.1)	
Multifocality			0.665^a^		0.515^a^
Yes	14 (33.3)	58 (36.9)		33 (39.1)	
No	28 (66.7)	99 (63.1)		51 (60.7)	
Histologic subtypes			0.673^a^		0.575^a^
Well-differentiated	9 (21.4)	42 (26.8)		25 (29.8)	
Dedifferentiated	28 (66.7)	102 (65)		50 (59.5)	
Myxoid/Round cell	2 (4.8)	7 (4.5)		6 (7.1)	
Pleomorphic	3 (7.1)	6 (3.8)		3 (3.6)	
FNCLCC grade			0.116^a^		0.186^a^
G1	7 (16.7)	41 (26.1)		23 (27.4)	
G2	24 (57.1)	61 (38.9)		34 (40.5)	
G3	11 (26.2)	55 (35)		27 (32.1)	
No. of organs resected [median (IQR)]	6 (4–8)	4 (3–7)	0.032^b^	5 (4–8)	0.068^b^

Values were shown as n (%), or median (interquartile range); ^a^ Comparison of data using the two-sided Chi-square test; ^b^ Comparison of data using the Mann-Whitney U test

### Surgical details and postoperative outcomes

As shown in Table 2, the type of vessels resected were as following: 21 combined iliac artery and vein, 15 inferior vena cava (IVC), 4 iliac vein and 2 iliac artery. 92.8% of the patients underwent segmental resection with prosthetic reconstruction, whereas 3 patients received side-wall resection. At the final pathology, the resected vessels were infiltrated by the RLPS in 27 (64%) patients. 97.6% (41/42) of the patients obtained R0 margin. At a median follow up of 48 months, 86% of the resected vessels remained patent. The colon, gallbladder, kidney, psoas major muscles, and adrenal gland were the most commonly resected organs in the vascular resection group.

**Table 2:** Operative characteristics of retroperitoneal liposarcoma (RLPS) resection en-bloc with the major vessels

Variables	RLPS & the major vascular resection (*n* = 42)
Vessels resected, n (%)	
Inferior vena cava	15 (35.8%)
Iliac vein	4 (9.5%)
Iliac artery	2 (4.7%)
Iliac artery and vein	21 (50%)
Resected type, n (%)	
Side-wall resection	3 (7.2)
Segmental resection with reconstruction	39 (92.8)
Vascular infiltration, n (%)	
Yes	27 (64%)
No	15 (36%)
Margin of resected vessels, n (%)	
R0	41 (97.6%)
R1	1 (2.4%)
Vascular patency^a^, n (%)	36 (86%)
Additional resected organs, n (%)	
Liver	5 (11.9%)
Pancreas	17 (40.5%)
Spleen	6 (14.3%)
Gallbladder	27 (64.3%)
Adrenal gland	21 (50%)
Kidney	26 (61.9%)
Stomach	16 (38.1%)
Small bowel	19 (45.2%)
Colon	33 (78.5%)
Bladder	7 (16.7%)
Diaphragm	15 (35.7%)
Psoas major muscle	22 (52.4%)

^a^At the median follow up of 48 months

As shown in Table 3, the vascular resection group had longer operative time (570 vs. 420 min, *p* < 0.001), longer postoperative hospital stay (26 vs. 20 days, *p* = 0.004), and greater estimated blood loss (2800 vs. 800 ml, *p* < 0.001) in the entire cohort. 38 (19.1%) patients experienced a major complication, with a higher incidence in the vascular resection group (38% vs. 14%, *p* < 0.001). Venous thromboembolism (VTE) (14.3%), postoperative abdominal bleeding (9.5%) and abdominal infection (7.1%) were the most common major complications in the vascular resection group. Reoperation was required in 19 (9.5%) patients, with a greater incidence in the vascular resection group (19% vs. 7%, *p* = 0.017). 5 (2.5%) patients died within 30 days after surgery and higher mortality was observed in the vascular resection group (7.1% vs. 1.3%, *p* = 0.005). In the matched cohort, the results are similar.

**Table 3:** Surgical details and postoperative outcomes before and after propensity score matching

Variables	Vascular resection (*n* = 42)	Before matching		After matching	
		Non-vascular resection (*n* = 157)	*P* value	Non-vascular resection (*n* = 84)	*P* value
Operative time (min)	570 (445–692)	420 (300–547)	< 0.001	444 (305–586)	< 0.001
Estimated blood loss (ml)	2800 (1750–5500)	800 (300–2000)	< 0.001	800 (400–2425)	< 0.001
Postoperative hospital stay (day)	26 (18–34)	20 (14–27)	0.004	20 (13–28)	0.005
Pancreatic resection, n (%)	18 (42.9%)	79 (50.3%)	0.390	33 (39.3%)	0.700
Nephrectomy, n (%)	27 (64.3%)	100 (63.7%)	0.944	49 (58.3%)	0.520
Major complications, n (%)	16 (38%)	22 (14%)	< 0.001	12 (14.3%)	< 0.001
Postoperative abdominal bleeding	4 (9.5%)	6 (3.8%)		3 (3.6%)	
Abdominal infection	3 (7.1%)	4 (2.4%)		2 (2.3%)	
Gastrointestinal anastomotic leak	1 (2.4%)	5 (3.1%)		3 (3.6%)	
Bowel obstruction	0 (0)	1 (0.6%)		1 (1.2%)	
VTE	6 (14.3%)	1 (0.6%)		0 (0)	
Renal insufficiency	1 (2.4%)	0 (0)		0 (0)	
Postoperative pancreatic fistula	1 (2.4%)	5 (3.2)		3 (3.6%)	
Reoperation, n (%)	8 (19%)	11 (7%)	0.017	6 (7.1%)	0.020
Postoperative abdominal bleeding	3 (9.5%)	4 (2.5%)		2 (2.3%)	
Abdominal infection	2 (4.8%)	2 (1.8%)		2 (2.3%)	
Gastrointestinal anastomotic leak	1 (2.4%)	4 (2.5%)		1 (1.2%)	
Bowel obstruction	0 (0)	1 (0.6%)		1 (1.2%)	
VTE	2 (4.8%)	0 (0)		0 (0)	
Death within 30 days of surgery, n (%)	3 (7.1%)	2 (1.3%)	0.005	1 (1.2%)	0.016

Abbreviations: VTE, venous thromboembolism

**Fig. 1:**
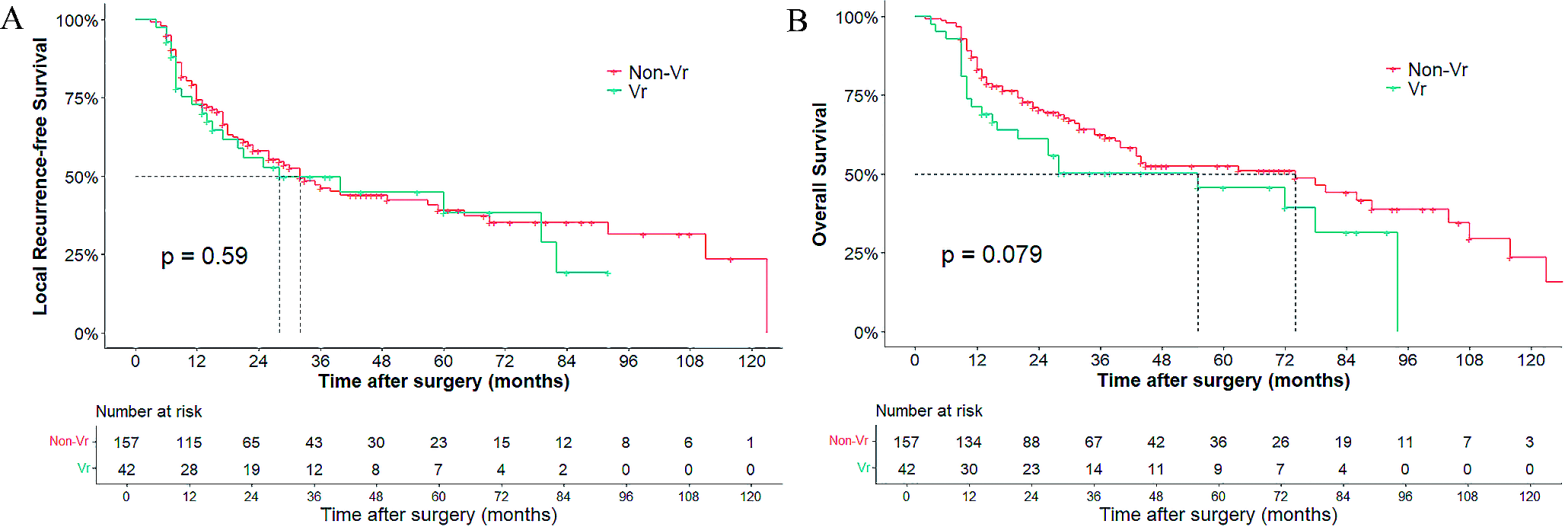
Kaplan-Meier curve for local recurrence-free survival (**A**) and overall survival (**B**) in the entire cohort before Propensity Score Matching Analysis. Vr, Vascular resection group; non-Vr, non-Vascular resection group

**Fig. 2:**
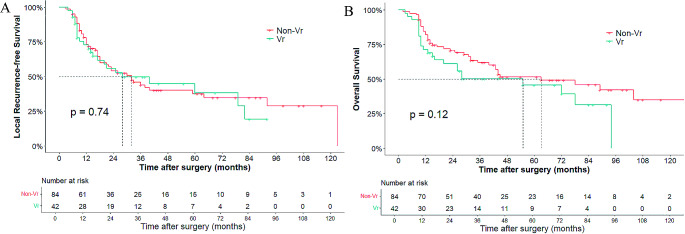
Kaplan-Meier curve for local recurrence-free survival (**A**) and overall survival (**B**) in the matched cohort after Propensity Score Matching Analysis. Vr, Vascular resection group; non-Vr, non-Vascular resection group

The median follow-up period was 48 (IQR 45–69) months in the entire cohort and 61 (IQR 48–73) months in the matched cohort. The estimated 5-year LRFS and OS rates in the entire cohort were 37.9% and 47.3%, respectively. The median LRFS and OS in the vascular resection group were 28 and 55 months, whereas that in the non-vascular resection group were 32 and 74 months. No significant difference was observed in survival rates between the vascular resection and non-vascular resection groups (LRFS: 38.4% vs. 37.2%, *P* = 0.59, Fig. 1A; OS: 45.7% vs. 50.9%, *P* = 0.079, Fig. 1B). After the propensity-matched analysis, the corresponding 5-year LRFS rates of the vascular resection and non‐vascular resection groups were 36.5% and 34.7%, with a median LRFS of 28 and 32 months, respectively (*p* = 0.74). The 5‐year OS rates of the vascular resection and non‐vascular resection groups were 39.2% and 49%, with a median OS of 55 and 63 months, respectively (*p* = 0.12). The LRFS and OS curves of the matched cohort are shown in Fig. 2A and B.

### Multivariate analysis for LRFS and OS

The independent risk factors of LRFS and OS in the matched cohort are presented in Table 4. Factors including presentation, histological subtype, FNCLCC grade, location and major vascular resection were included in the multivariate analysis of LRFS. Location (*p* = 0.004) and FNCLCC grade (*p* = 0.032) were independent factors that influenced LRFS. Factors including size, histological subtype, FNCLCC grade, and the number of organs resected were incorporated into the multivariate analysis for OS. Larger tumor (*p* = 0.015) and increased number of organs resected (*p* = 0.034) were independently associated with inferior OS.

**Table 4:** Multivariate analysis of survival between vascular resection and non-vascular resection groups after propensity score matching

Variables	Local recurrence-free survival		Overall survival	
	HR (95%CI)	*P* value	HR (95% CI)	*P* value
Presentation (recurrence vs. primary)	1.218 (0.711–2.084)	0.473	—	—
Tumor size	—	—	1.039 (1.006–1.070)	**0.015**
Histological subtypes		0.314		0.121
DD vs. WD	3.585 (0.945–13.598)		4.609 (0.904–23.502)	
Myxoid/Round Cell vs. WD	3.617 (0.769–17.008)		7.854 (1.445–42.693)	
Pleomorphic vs. WD	3.355 (0.617–18.249)		5.029 (0.776–32.528)	
FNCLCC grade		**0.032**		0.278
G2 vs. G1	1.896 (0.235–3.418)		2.070 (0.350-12.256)	—
G3 vs. G1	4.246 (2.302–9.138)		2.988 (0.475–18.777)	—
Location		**0.004**		
Abdominopelvic vs. Abdominal	2.557 (1.474–4.436)		—	—
Pelvic vs. Abdominal	1.234 (0.462–3.296)		—	—
Major vascular resection (yes vs. no)	0.958 (0.568–1.615)	0.872	—	—
No. of organs resected	—	—	1.116 (1.042–1.321)	**0.034**

Abbreviations: WD, well-differentiated; DD, dedifferentiated; FNCLCC, French National Federation of the Centers for the Fight Against Cancer; 95%CI, 95% confidence interval; HR, hazard ratio

Bold indicated the data was statistically significant

## Discussion

Multi-visceral resection of RLPS may necessitate vascular resection and reconstruction(Tzanis et al. 2018). Vascular invasion can be a sign of intrinsic biological aggressiveness (Devaud et al. 2023). In our cohort, the 5-year LRFS and OS rates were similar between patients with and without the vascular resection. This reflects that major vascular involvement does not represent a contraindication for curative resection of RLPS. To our knowledge, the present study is one of the largest studies solely focused on homogeneous RLPS to assess surgical outcomes of major vascular resection by using a propensity score matching analysis.

Liposarcoma and leiomyosarcoma represent the predominant histologic subtypes of RPS (de Bree et al. 2023). Major vascular resection in surgery for RLPS is less infrequent compared with retroperitoneal leiomyosarcoma (Devaud et al. 2023). Thanks to the improvements in the surgical techniques and perioperative management, en-bloc resections with the invaded vessels are no longer a contraindication to surgery for RLPS (Fiore et al. 2012). As one of the largest specialized sarcoma centers in China, we have performed 42 (21%) major vessel resection for RLPS in 199 patients. The frequency was similar to that reported by the University of Heidelberg (17.7%) (Schwarzbach et al. 2006), but is higher than those reported by Spolverato et al. (5%) (Spolverato et al. 2021). This could be attributed to the fact that the decision to perform major vessel resection may be affected by various factors, including different surgical strategies and the surgeon’s familiarity with the technique of vascular resection. In our study, patients with recurrent tumors underwent previous simple resection instead of the extended resection in other hospitals, nearly all of which were not high-volume centers. Previous studies have shown that re-resection for tumor residue after initial inadequate resection could provide a similar overall survival compared to complete primary resection (Nizri et al. 2019). Therefore, we performed relatively aggressive surgical treatment for patients with recurrent tumors after the initial simple resection. This is the reason why 48% of the patients had recurrent tumors and 33% presented with multifocality in the vascular resection group. In the present study, vascular infiltration was confirmed in 27 (64%) patients and R0 resection was achieved in 80.4% of the patients. This is in line with the vascular infiltration rate (70%) reported by Fairweather et al. (Fairweather et al. 2018). Therefore, adjacent major vessels should be resected when they are widely encased or frankly infiltrated.

Different reconstructive techniques have been developed over the past decades and are not standardized (Dull et al. 2013). The inferior vena cava and iliac vessels were the most commonly involved vessels in RPS resection (Quinones-Baldrich et al. 2012). Moreover, both venous and arterial resection were commonly required in cases of RPS involving iliac vessels (Radaelli et al. 2016). The methods of reconstruction include partial resection, primary repair, transplantation, and ligation (Quinones-Baldrich and Farley 2013). Reconstructions with PTFE grafts are the most reported reconstructive technique in published articles (Blair et al. 2018; Quinones-Baldrich et al. 2012). Similarly, PTFE grafts were used in the majority of the patients in our cohort. Of note, the graft patency rates (86%) were comparable to those in other studies that reported a graft patency of 80-90% (Kieffer et al. 2006; Shafique et al. 2024). Previous reports have associated a higher risk of VTE with vascular reconstruction (Blair et al. 2018). Indeed, VTE (14.3%) was the leading cause of major morbidity and reoperations in the vascular resection group, followed by postoperative abdominal bleeding and abdominal infection.

Vascular resection increases the complexity of the operation, which involve high risk (Devaud et al. 2023). Different retrospective studies have confirmed that risk of RPS surgery are higher in patients who underwent major vascular resection, with the major morbidity and mortality rates of approximately 18-50% and 0-21% (Devaud et al. 2023), respectively. In a France study analyzing 31 patients with RPS who received oncovascular surgery, the reoperation rate was 16% (Bertrand et al. 2016), which is consistent with our current finding of a reoperation rate of 19%. In another retrospective analysis, the major morbidity and mortality rates of surgery for PRS involving major vessels were 36% and 4%, respectively (Schwarzbach et al. 2006). Similarly, in this series of 42 patients with en-bloc vascular resection, the major complication and mortality rates were 38% and 7.1%, respectively. Given the great number of organs resected, intrinsic aggressiveness, and malnutrition, the high morbidity, reoperation and mortality rates were feasible in the vascular resection cohort. Thus, meticulous preoperative planning and resection policy should be adopted when major vascular structures are involved in patients with RLPS.

In terms of the high morbidity and mortality after extensive resections with the invaded vessels, downstaging RPS with neoadjuvant therapy to facilitate adequate resection is crucial. But the role of perioperative therapy remains controversial for RPS (de Bree et al. 2023). The global randomized trial (STRASS) to evaluate the role of surgery with and without preoperative RT for patients with RPS, did not demonstrate a significant overall benefit (Bonvalot et al. 2020). RLPS was less sensitive to conventional doxorubicin-based chemotherapy (Italiano et al. 2012). The benefit of neoadjuvant chemotherapy remains unclear from retrospective data and await confirmation by the ongoing STRASS2 randomized controlled trial (Lambdin et al. 2023). Due to the unsatisfactory effect of perioperative therapy seen in the most RPS cases, administration of systemic therapies or radiotherapy in neoadjuvant and adjuvant setting is not the current standard of care (de Bree et al. 2023). Further randomised trials are warranted to identify the definite role of systemic therapies or radiotherapy in the multimodality treatment of RPS.

Studies have shown that survival rates of patients who underwent major vascular resection are comparable to those without vascular involvement (Blair et al. 2018; Poultsides et al. 2015). Our study showed similar 5-year LRFS and OS rates between patients with and without the vascular resection in the matched cohort (LRFS: *P* = 0.74, OS: *p* = 0.12, respectively). This finding was comparable to those reported by Hu et al. (Hu et al. 2023) and Blair et al. (Blair et al. 2018). Vascular resection was also not an independent predictor influencing LRFS and OS in patients with RLPS. Multivariate analyses confirmed that larger tumor size and higher numbers of organs resected are independent adverse prognostic factors of OS. It could be inferred that the necessity of increased number of organs resected may imply more aggressive tumor behavior and high difficulty of tumor removal. As aforementioned, the need of vascular resection for RLPS is not associated with a worse oncological prognosis.

Taken together, the study offers a valuable perspective for the role of major vascular resection in the surgical management of RLPS. Nevertheless, our study had several limitations. First, this study was performed retrospectively and the inherent selection bias could not be avoided. Second, the sample size of patients undergoing vascular resection was limited and the criteria for vascular resection was not prospectively selected. Finally, the assessment of long-term vascular patency was insufficient and longer follow-up is needed. Further studies from multi-institutional and international collaborations are needed to verify these results over a longer time span.

## Conclusions

Although accompanied by increased risks of major morbidity and mortality, the major vascular resection enabled radical resection in patients with advanced RLPS, affording comparable 5-year LRFS and OS rates compared to those without vascular involvement. Major vascular involvement does not represent a contraindication for curative resection of RLPS. When clinically indicated, major vascular resection should be performed cautiously at specialized high-volume centers after weighing the oncological benefits and the risk of severe morbidity.

## Data Availability

No datasets were generated or analysed during the current study.
